# Primary tuberculous liver abscess in an immunocompromised patient: a case report

**DOI:** 10.1099/acmi.0.001080.v3

**Published:** 2026-09-15

**Authors:** Imane Bentaher, Fatna Bssaibis, Yassine Ben Lahlou, Elmostafa Benaissa, Mariama Chadli

**Affiliations:** 1 Bacteriology Laboratory, Mohammed V Military Teaching Hospital, Rabat, Morocco; 2 Faculty of Medicine and Pharmacy of Rabat, Rabat, Morocco

**Keywords:** isolated abscess, liver, primary, tuberculosis

## Abstract

Isolated tuberculous abscess of the liver is an extremely rare clinical entity and represents a diagnostic challenge, as it can easily be confused with other hepatic lesions of infectious or tumoral origin. We report the case of a 67-year-old immunocompromised female patient with no known history of tuberculosis who was admitted for a liver abscess initially detected by abdominal computed tomography scan. Aspiration of the lesion revealed an infection involving *Mycobacterium tuberculosis*, confirmed by PCR analysis. The patient was treated with an appropriate antibiotic regimen of standard four-drug antituberculous therapy. Clinical and radiological follow-up showed progressive improvement, with complete recovery achieved after 6 months of treatment. This case highlights the importance of considering hepatic tuberculosis, particularly in endemic regions and in immunocompromised individuals.

## Data Summary

No data were reused or generated in this study.

## Introduction

Tuberculosis is a major public health issue in Morocco and ranks among the ten leading causes of death worldwide. The World Health Organization (WHO) classifies Morocco as a country with medium incidence, with 35,000 new cases in 2021 [1]. While the pulmonary form remains the most common, extrapulmonary forms are not uncommon, accounting for ∼15–20% of cases, with a rate of up to 50% in immunocompromised individuals [2, 3]. Among these, liver involvement is uncommon, occurring most often in the context of disseminated miliary tuberculosis and rarely in isolation [2, 4].

Isolated tuberculous liver abscess, without associated pulmonary or digestive involvement, is an even more exceptional form, accounting for less than 0.3% of extrapulmonary tuberculosis (EPTB) [2]. Its diagnosis is particularly complex: the clinical signs are nonspecific, the pathology is rare and the level of suspicion often remains low [3, 5, 6]. This explains why this type of abscess is frequently confused with pyogenic or amoebic liver abscesses, or even with a necrotic tumour [3, 7].

In this context, we present a rare case of primary tuberculous liver abscess in a 67-year-old diabetic patient, revealed by molecular biology and confirmed by culture, illustrating the diagnostic challenges posed by this little-known entity, but also the importance of molecular biology techniques in the early diagnosis and appropriate management of these atypical forms.

## Case report

This is a 67-year-old female patient with a history of type II diabetes treated with insulin, end-stage chronic renal failure requiring chronic haemodialysis (10 years) due to polycystic kidney disease, hypothyroidism treated with levothyroxine for 2 years, non-ischaemic heart disease with severe left ventricular dysfunction (20%) and right atrial thrombus treated with nicoumalone and chronic diverticulitis with a tendency to secondary infection treated with medication. The patient reports no documented history of pulmonary tuberculosis (sputum examination by Ziehl–Neelsen staining, culture on Lowenstein–Jensen^®^ (LJ^®^) solid medium and PCR using GeneXpert MTB/RIF^®^ on three consecutive days returned negative results 1 year ago), no infection and no signs of impregnation. Her Bacillus Calmette–Guérin vaccination status is unknown.

She was admitted to the emergency room for treatment of right upper quadrant pain associated with vomiting and effective transit. Physical examination revealed that the patient was conscious, apyretic, anicteric and had normal vital signs. Abdominal examination revealed a soft abdomen, tenderness in the right hypochondrium and no hepatomegaly, all in the context of a stable general condition.

Her blood tests showed a complete blood count with haemoglobin at 9.7 g dl^−1^, haematocrit at 52%, leukocytes at 7,200 cells mm^−3^, of which 74.8% were neutrophils and platelets at 342,000 µl^−1^, prothrombin time (PT) at 52%, an activated partial thromboplastin time of 52 s and a C-reactive protein (CRP) level decreasing from 270 to 74 mg l^−1^. Her liver function tests were normal with no cholestasis or cytolysis: total bilirubin 4 mg l^−1^, direct bilirubin 3 mg l^−1^, γ-glutamyl transferase (GGT) at 30 U l^−1^, alkaline phosphatase at 107 U l^−1^, alanine aminotransferase (ALAT) at 5 IU l^−1^ and aspartate aminotransferase (ASAT) at 29 IU l^−1^; alkaline reserves at 19 mmol l^−1^. Human immunodeficiency virus (HIV) serology was negative (Table 1). No chest X-ray was performed.

**Table 1.** Table summarizing the patient’s laboratory results

Parameter	Result
**Haematology**	
Haemoglobin	9.7 g dl^−1^
Hematocrit	52 %
Leukocytes	7,200 mm^−³^
Neutrophils	74.8 %
Platelets	342,000 µl^−1^
**Coagulation**	
PT	52 %
Activated partial thromboplastin time	52 s
**Inflammatory marker**	
CRP	270 → 74 mg l^−1^
**Liver function tests**	
Total bilirubin	4 mg l^−1^
Direct bilirubin	3 mg l^−1^
GGT	30 Ul^−1^
Alkaline phosphatase	107 Ul^−1^
ALAT	5 IU l^−1^
ASAT	29 IU l^−1^
**Alkaline reserves**	19 mmol l^−1^
**HIV serology**	Negative

**Fig. 1.**
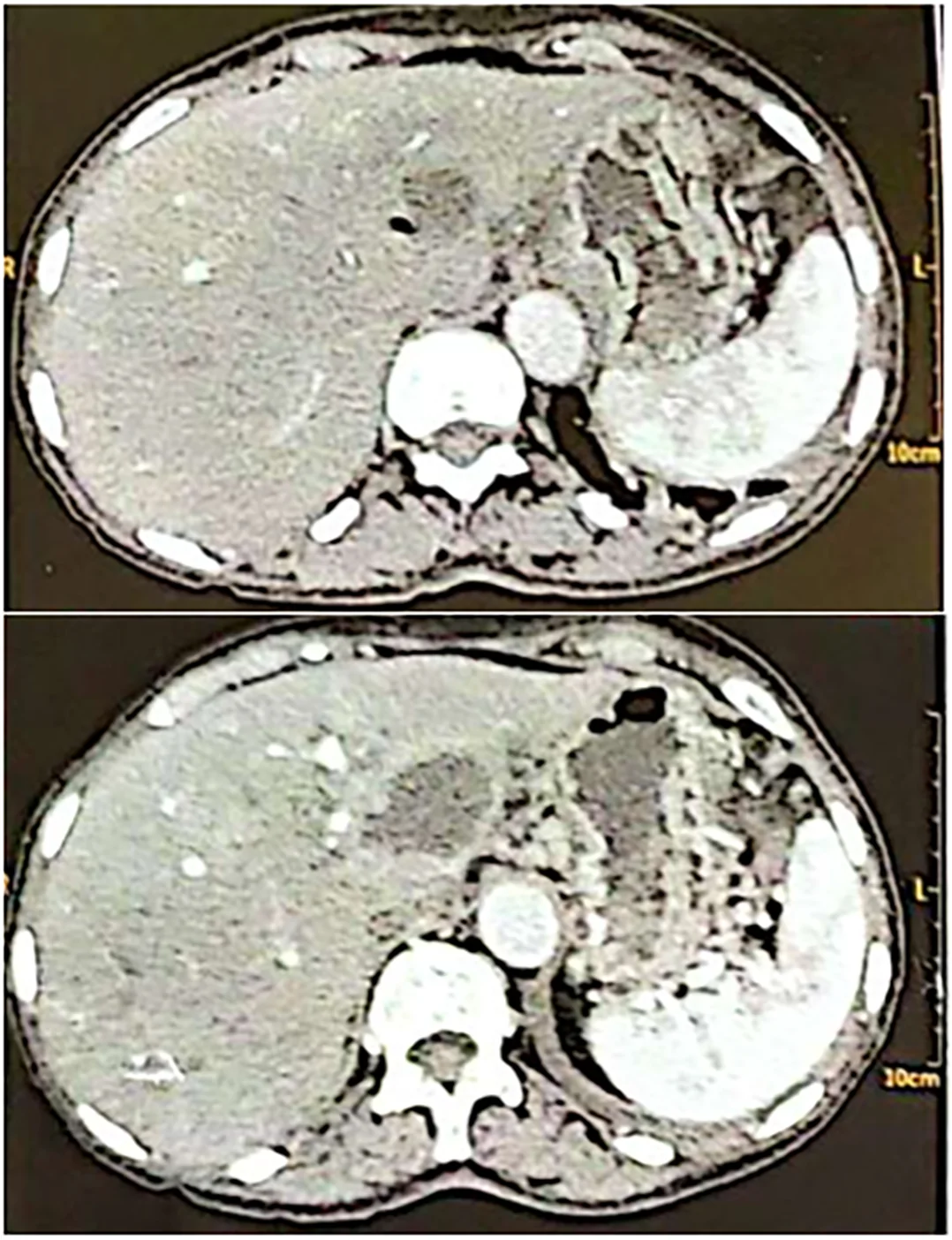
Axial abdominal CT scan with contrast injection.

Her abdominal computed tomography (CT) scan revealed a fluid collection containing air bubbles straddling segments I and II with enhanced walls after contrast injection, measuring 54×21 mm, peri-vesicular oedema and no portal thrombosis, all consistent with a hepatic abscess straddling segments II and I (Fig. 1). She then underwent abscess puncture (due to a 2 cm decrease in abscess volume 5 days after admission) and initial probabilistic antibiotic treatment (fluoroquinolones and metronidazole).

The sample from the puncture was sent to the bacteriology laboratory. It was haemopurulent in appearance and its routine culture, after grinding and sonication, was sterile. A search for the *Mycobacterium tuberculosis* complex in the sample proved positive using molecular and conventional methods. Molecular analysis using the GeneXpert MTB/RIF^®^ test (Cepheid, Sunnyvale, CA, USA), which uses automated real-time PCR (RT-PCR), revealed the presence of *M. tuberculosis* complex (low load) without detection of rifampicin resistance.

**Fig. 2.**
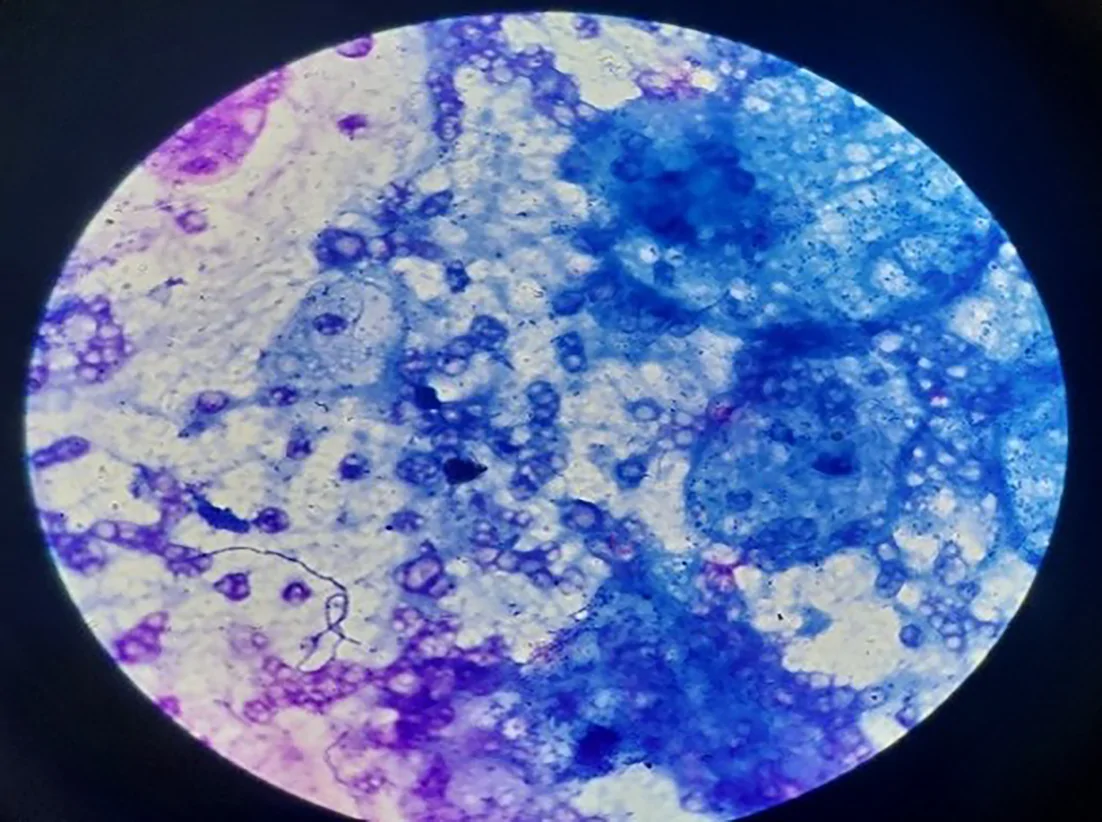
Ziehl–Neelsen staining – microscopic observation at 100× magnification (immersion) of AFB.

Direct examination after preliminary staining with auramine and then by special Ziehl–Neelsen staining was positive [1–10 acid-fast Bacilli (AFB)/100 fields] (Fig. 2). Cultures on LJ^®^ solid medium and Mycobacteria Growth Indicator Tube (MGIT^®^) liquid medium became positive after 58 and 45 days, respectively, confirming the molecular diagnosis. No liver biopsy was performed for pathological examination.

As part of the additional workup, sputum testing for tuberculosis was performed on three consecutive days using the same methods and was negative for *M. tuberculosis* complex.

Evidence of a hypodense fluid collection enhanced after injection, straddling segments II and I of the liver.

The patient was placed on first-line quadruple therapy with oral antibacillary drugs (2RHZE/4RH) (R: Rifampicin 600 mg; H: Isoniazid 300 mg; Z: Pyrazinamide 1,000 mg and E: Ethambutol 1,500 mg). Four weeks later, the patient was asymptomatic. A follow-up ultrasound scan at 2 months confirmed the positive progress, showing regression of the liver abscess. The outcome was favourable, with recovery after 6 months.

## Discussion

EPTB accounts for ∼15–20% of all tuberculosis cases in immunocompetent patients. In patients with HIV or other immunocompromising conditions, this prevalence increases significantly, up to 50% or more of all tuberculosis cases [2, 3, 8, 9]. Within EPTB, abdominal tuberculosis represents ∼3–5% of extrapulmonary cases in developed countries but may reach 11–16% in endemic regions [8, 10]. Liver involvement is common in disseminated forms of tuberculosis, occurring in 50–80% of cases of miliary tuberculosis. However, the occurrence of a tuberculous liver abscess without pulmonary or digestive involvement remains rare [2–5], with an estimated prevalence of only 0.34% among cases of hepatic tuberculosis [7, 9].

The isolated form is most often seen in immunocompromised patients [3], such as our patient. Certain ethnic groups also appear to be at higher risk, particularly children, African Americans and Igorots [11].

This diagnosis is probably underestimated due to the frequent absence of specific clinical symptoms, which contributes to its low detection rate in routine practice [5, 12].

Diagnosing hepatic tuberculosis remains a real challenge for clinicians. Its non-specific clinical presentation and variable radiological images explain why it is often confused with other liver diseases, such as hepatocellular carcinoma, amoebic liver abscess or pyogenic abscess [2, 3, 5–7]. Several published cases illustrate how patients are initially treated for these alternative diagnoses before tuberculosis is finally confirmed [3, 7, 11]. In many cases, the diagnosis is only made during surgery, during a laparotomy or even after death, at autopsy [3, 6, 7, 9, 11]. Traditional microbiological tools add to the difficulty: cultures take weeks to turn positive and are frequently negative in paucibacillary disease, while histology may be inconclusive in the absence of characteristic caseating granulomas [12, 13]. These limitations explain why many cases are recognized only late in the course of illness. In recent years, molecular techniques, particularly PCR, have helped to shorten this diagnostic delay by offering rapid and highly specific confirmation of *M. tuberculosis* [2, 10, 13]. Ultimately, the best chance for timely diagnosis lies in combining careful clinical assessment with imaging, pathology and molecular testing, while maintaining a high level of suspicion in endemic areas.

Three main forms of hepatic tuberculosis have been described in the literature. Here, we adopt the classification proposed by Reed, which distinguishes between: (1) liver involvement in the context of disseminated miliary tuberculosis, (2) primary miliary tuberculosis of the liver and (3) hepatic tuberculoma or primary tuberculous abscess [5, 11].

Tubercle bacilli reach the liver via the bloodstream: via the hepatic artery in miliary forms and via the portal vein in localized forms [9]. Thanks to its rich vascularization and the abundant presence of cells from the reticuloendothelial system, the liver is a favourable environment for the formation of granulomas, which are generally located around the portal spaces. Despite this infiltration, liver function often remains largely unaffected, which explains why the disease frequently progresses discreetly, with few or no symptoms. The primary form of hepatic tuberculosis remains rare, particularly due to the low oxygen content of liver tissue, an environment that is not conducive to the proliferation of *M. tuberculosis* [5].

Hepatic tuberculosis most often manifests with nonspecific clinical signs, which complicates diagnosis. The most frequently reported symptoms are fever, general malaise, pain in the right upper quadrant, as in the case of our patient, night sweats and sometimes hepatomegaly [2–5, 9, 11]. Jaundice is rare and usually occurs when there is significant tuberculous infiltration or obstruction of the bile ducts [2, 5]. However, there is no clear link between the severity of liver damage and the onset of jaundice [7, 9].

Biologically, elevated alkaline phosphatase levels are frequently observed, sometimes accompanied by moderate cholestasis. Transaminases, on the other hand, are generally only slightly elevated [3–5, 11]. Blood counts may reveal anaemia, as in the case of our patient and hyperleukocytosis [8, 14]. Other biological abnormalities are sometimes found, such as hyponatraemia, prolonged PT or an inverted albumin/globulin ratio [3].

On ultrasound, the macronodular form generally presents as one or more hypoechoic masses, without posterior enhancement, sometimes punctuated by calcifications [7, 8, 11]. On CT scan, hepatic tuberculous abscess appears as a hypodense lesion with polylobulated contours and moderate peripheral enhancement, which may suggest a pyogenic abscess [7]. Magnetic Resonance Imaging (MRI) allows for a more detailed analysis, particularly according to histopathological stages [4, 5].

The microbiological diagnosis of hepatic tuberculosis is based mainly on the detection of *M. tuberculosis* in aspirated pus or from a liver biopsy using several methods: AFB testing, culture and RT-PCR [2, 7, 9, 11]. Ziehl–Neelsen staining, although simple to perform, lacks sensitivity. As for culture, which remains the gold standard, it takes a long time to obtain results and is often negative, particularly in paucibacillary forms [2, 11], which is consistent with the delayed positivity of our patient’s cultures. However, in areas of high endemicity, such as Morocco, the absence of bacilli should not rule out the diagnosis [7, 12].

PCR is currently the most widely used technique for diagnosing hepatic tuberculosis [2, 10, 15]. It has excellent sensitivity (∼92%) and specificity greater than 98%, with positivity rates well above those of conventional methods – up to 88% according to Alcantara–Payawal and 57% according to C.P. Baveja [2, 9, 16]. In addition to being rapid, PCR also makes it possible to differentiate *M. tuberculosis* from other mycobacteria, which greatly facilitates therapeutic management [10, 13]; in fact, it enabled early management of our case.

Histological examination of the tissue samples, particularly from the abscess wall, confirms the diagnosis. A typical tuberculous granuloma with central caseous necrosis is usually found [7].

Treatment of hepatic tuberculosis is based on prolonged antituberculous antibiotic therapy. The classic four-drug regimen – isoniazid, rifampicin, pyrazinamide and ethambutol – remains the basis of treatment. A good clinical response often makes it possible to avoid surgery, as evidenced by the marked regression of the abscess in our patient under medical treatment alone. However, when the abscess is large or surrounded by a thick fibrous capsule, antibiotic penetration may be limited. In such cases, image-guided percutaneous drainage, combined with local instillation of anti-tuberculosis drugs, can significantly improve the response to treatment, while being less invasive than surgery. Surgery is, therefore, reserved for complex situations or in cases where percutaneous drainage has failed [2, 3, 5, 7, 17].

Therapeutic monitoring of a tuberculous liver abscess requires, for at least 6 months, combined clinical, biological and radiological follow-up. Clinically, symptoms such as fever, abdominal pain and general condition should be tracked to evaluate treatment response and detect complications [1–3, 7, 10]. Liver function tests are essential due to the hepatotoxicity of first-line drugs, and vigilance is especially important in immunocompromised patients, who may present with atypical or severe courses [1, 5, 6, 11, 16]. Imaging, mainly ultrasound and CT, is central to assess abscess regression, as was the case of our patient, and to decide on percutaneous or surgical drainage if needed [3, 12, 14, 17]. Bacteriological tests like PCR or culture can support confirmation of clearance [16]. Long treatment duration and drug-related side effects make adherence and management of drug interactions, particularly in immunocompromised patients, a critical part of monitoring [2, 5, 11, 15].

## Conclusion

Hepatic tuberculosis, although uncommon, should always be considered when a liver mass or cystic lesion is discovered, particularly in endemic regions or in immunocompromised patients. The challenge lies in its nonspecific presentation, which often resembles pyogenic or amoebic abscesses or even malignant lesions, leading to delayed or missed diagnoses. To improve outcomes in similar cases, we recommend maintaining a high index of suspicion, especially when first-line treatments fail. A structured diagnostic approach is essential: careful imaging to characterize the lesion, aspiration whenever feasible and systematic microbiological investigations. RT-PCR offers rapid and highly sensitive confirmation, while Ziehl–Neelsen staining and culture provide additional reliability when positive. Integrating these methods ensures greater diagnostic accuracy, allowing earlier initiation of therapy. Prompt recognition and timely antituberculous treatment remain the cornerstone for recovery, avoiding unnecessary invasive procedures and improving prognosis in these rare but serious presentations.

## Abbreviations

AFB: acid-fast bacilli
ALAT: alanine aminotransferase
ASAT: aspartate aminotransferase
CRP: C-reactive protein
CT: computed tomography
EPTB: extrapulmonary tuberculosis
GGT: γ-glutamyl transferase
HIV: human immunodeficiency virus
LJ®: Lowenstein–Jensen®
MGIT: Mycobacteria Growth Indicator Tube
MRI: Magnetic Resonance Imaging
PCR: polymerase chain reaction
PT: prothrombin time
RT-PCR: real-time PCR
WHO: World Health Organization
